# A three-dimensional, population-based average of the C57BL/6 mouse brain from DAPI-stained coronal slices

**DOI:** 10.1038/s41597-020-0570-z

**Published:** 2020-07-13

**Authors:** Frederik Filip Stæger, Kristian Nygaard Mortensen, Malthe Skytte Nordentoft Nielsen, Björn Sigurdsson, Louis Krog Kaufmann, Hajime Hirase, Maiken Nedergaard

**Affiliations:** 1 Center for Translational Neuromedicine, Faculty of Health and Medical Sciences, University of Copenhagen, 2200 Copenhagen, Denmark; 2 Center for Translational Neuromedicine, University of Rochester Medical Center, Rochester, NY 14642 USA

**Keywords:** Computational neuroscience, Image processing

## Abstract

Fluorescence imaging of immunolabeled brain slices is a key tool in neuroscience that enable mapping of proteins or DNA/RNA at resolutions not possible with non-invasive techniques, including magnetic resonance or nuclear imaging. The signal in specific regions is usually quantified after manually drawing regions of interest, risking operator-bias. Automated segmentation methods avoid this risk but require multi-sample average atlases with similar image contrast as the images to be analyzed. We here present the first population-based average atlas of the C57BL/6 mouse brain constructed from brain sections labeled with the fluorescence nuclear stain DAPI. The data set constitutes a rich three-dimensional representation of the average mouse brain in the DAPI staining modality reconstructed from coronal slices and includes an automatic segmentation/spatial normalization pipeline for novel coronal slices. It constitutes the final population-based average template, individual reconstructed brain volumes, and native coronal slices. The comprehensive data set and accompanying spatial normalization/segmentation software are provided. We encourage the community to utilize it to improve and validate methods for automated brain slice analysis.

## Abstract (summary)

**Table:** 

Measurement(s)	brain measurement
Technology Type(s)	epifluorescence microscopy • magnetic resonance imaging (MRI)
Factor Type(s)	brain region
Sample Characteristic - Organism	Mus musculus • C57BL/6 mouse strain

Machine-accessible metadata file describing the reported data: 10.6084/m9.figshare.12554666

## Background & Summary

Biological studies routinely assess the abundance and localization of specific proteins or DNA/RNAs within complex tissues using an array of techniques. RNA expression can be visualized in tissue sections with single-stranded probes tagged with a fluorophore, which bind to complementary strands of nucleic acids, whereas proteins are typically visualized by immunohistochemical or fluorescent methods using green-fluorescent-protein (GFP) or other genetically expressed fluorescent proteins. To accurately map the distribution of labelled RNA or protein within the tissue section, counterstaining is often utilized to identify anatomical landmarks. In the context of fluorescence microscopy, a classical choice of counterstain is nuclear stain 4′,6-diamidino-2-phenylindole (DAPI), which binds to the minor groove in the double-stranded DNA helix^1^. Due to the intricate methodology required for counterstaining and imaging, 3-dimensional data collected in whole rodent brains is most frequently displayed and analyzed in two-dimensional tissue sections.

Recent years have seen a significant increase in the use of mice as the preferred experimental model of diverse human diseases. The neuroanatomic and genetic similarity of mice to humans, in combination with their ease of breeding and the availability of a variety of transgenic reporter mice have contributed to the burgeoning popularity of mouse studies in neuroscience. In brain, protein expression patterns are evaluated in brain slices in one of the three planes, often the coronal plane. The intensity of the fluorescent or histochemical signal is analyzed in predefined regions of interest (ROIs) that are typically delineated manually by the investigator. However, manual analysis is time-consuming, imprecise, and prone to inter- and intra-operator variability. In contrast, human magnetic resonance imaging (MRI) anatomic analyses, and likewise studies in diverse species such as non-human primate^2^, pig^3^ or mouse are often based on either automatic segmentation using population-based atlases or by pixel-by-pixel comparisons after image registration to a common template^4–6^. Such approaches avoid operator variability and allow unbiased and detailed anatomic analysis but require high-quality registration target and well-tuned parameters. The particular choice of software and registration parameters will necessarily introduce some technical bias, but these are common to all experimental groups in the study and can be replicated in longitudinal studies, so long as treatments do not greatly perturb gross anatomy of the brain.

Manual identification and segmentation of mouse brain ROIs are typically carried out with reference to mouse brain atlases such as the Mouse Brain in Stereotaxic Coordinates^7^ and the Allen reference atlas^8^. Such atlases are commonly projected on acetylcholinesterase (AChE) or Nissl stained serial brain slices from a single representative specimen. To avoid bias towards the anatomy of the single specimen, the Allen Institute launched the Allen Mouse Common Coordinate Framework (CCF) version 3^8^, which is a population-based average template of 1675 specimens. However, the CCF was created using serial two-photon (STP) tomography, a modality which is unavailable in most experimental settings and yields tissue contrasts that do not always match the classical histochemical stains, such as DAPI. A mouse brain atlas delineated by DAPI staining would provide a useful anatomical reference for manual image analysis and would likewise facilitate automated image analysis. To our knowledge, neither single-subject nor population-averaged DAPI-based atlases have been available for adult mouse brain studies.

We here introduce a population based DAPI template reconstructed from 12 C57BL/6 mouse brains. This template enables automated registration and segmentation of DAPI-stained mouse brain slices. To construct the atlas, 12 mouse brains were cut into 100 µm coronal slices covering the majority of the cerebrum. These slices were DAPI-stained, imaged and reconstructed into three-dimensional volumes utilizing the Allen reference template. The volumes were then iteratively co-registered and restacked to create a final population-based average in the DAPI-stain modality. This DAPI template was then segmented by combining registrations to the Allen reference atlas and registration to a single-specimen Nissl-stained volume in the Allen reference space. We make available to the research community this DAPI template as well as our Python implementation of template-based coronal slice segmentation and spatial normalization. Additionally, we include *ex vivo* brain MR images, which were acquired to serve as an intact anatomical reference for each sample. The atlas consists of 62–70 serial 100-micron thick coronal brain slices from 12 mice, which are provided in the original, preprocessed, and reconstructed states.

## Methods

### Data acquisition

**Fig. 1:**
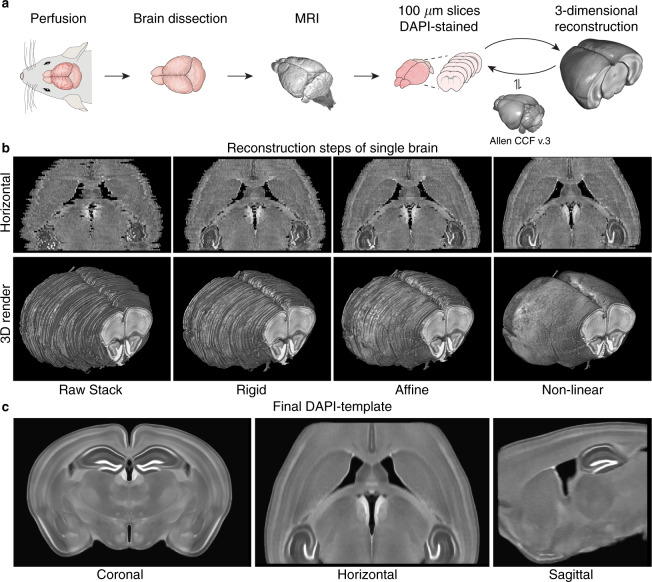
Overview of the DAPI template creation. (**a)** Schematic of the data acquisition and reconstruction procedure of each mouse: perfusion fixation, brain dissection, MRI, slicing and staining, imaging, and three-dimensional reconstruction, initially assisted by the Allen reference atlas (Allen CCF v.3). (**b)** Horizontal section and three-dimensional rendering of the initial stack and the three reconstruction steps for a single brain showing how the process approaches a smooth brain reconstruction with well-defined structures. (**c)** Representative cross-sections from the final population-based DAPI template in coronal, horizontal and sagittal planes.

Schematic overview in Fig. 1a. 12 male C57BL/6 mice aged between 10–11 weeks were anesthetized with a ketamine/xylazine mixture (0.015 mL/g of 9 mg/mL ketamine plus 0.2 mg/mL xylazine diluted in isotonic saline) and transcardially perfusion fixed with 4% paraformaldehyde (PFA). The brains were extracted from the skull, postfixed by immersion in 4% PFA overnight at 5 °C, and transferred to a medium containing 2.5 mM Magnevist (Gd-DTPA, BioPAL, Inc.) solution in phosphate-buffered saline which was allowed to permeate for two days at 5 °C. The brains were then scanned on a 9.4 T Bruker Biospec 94/30USR magnet, with a 3D-FLASH sequence (TR: 50 ms, TE: 12 ms, FA: 30°, FOV: 11 × 9 × 18 mm, matrix: 330 × 270 × 180) using a 23 mm volume coil. MRI scans were acquired to include intact anatomical reference images for each sample, but were not utilized in atlas construction *per se*. We include the MRI in the data set for completeness and encourage their use by other researchers. After imaging, each brain was cut into 100 µm thick coronal sections on a vibratome yielding 62 to 70 consecutive slices per brain covering the majority of the cerebrum excluding the olfactory bulb, the most posterior part of the hippocampus, and the most anterior and posterior parts of the neocortex. All slices were DAPI-stained (10 minutes in 1 µg/mL DAPI diluted in phosphate-buffered saline), mounted, and imaged on a Nikon (DS-Fi3) epifluorescence microscope, while ensuring proper left-right orientation. The images were acquired with a PlanApo Lambda 4X lens at 8-bit depth as a 5 × 5 grid automatically stitched together with 10% overlap resulting in 13248 × 9421 pixel images with pixel resolution of 0.86 × 0.86 µm. The acquired images were preprocessed by Otsu’s thresholding, largest connected-component extraction, normalization to the average pixel intensity in each slice and with N4-biasfield-correction^9^. The images were then down-sampled to 8.6 × 8.6 µm per pixel resolution and stored as single coronal slice images in the NIfTI-1 format for further processing.

### Volume reconstruction and template creation

The entire pipeline of volume reconstruction and template creation was run twice. In the first round, the images for each mouse brain were stacked into an initial three-dimensional brain volume. The Allen CCF average template^10^ was rigidly registered to this brain volume and resliced into 100 µm thick coronal two-dimensional images. Thus, each of the Allen slices corresponded to one of the DAPI brain slices. In each of the slice pairs, the brain slice was rigidly registered to the corresponding Allen slice. After this two-dimensional registration, the slices were restacked into a complete three-dimensional brain volume. This process was iterated three times with rigid body transformation and twice with affine registration, resulting in a set of well-aligned reconstructions of the brain volumes for the 12 mice.

A population-based template of the reconstructed volumes was created by iterative averaging and co-registering the reconstructed volumes, as previously described^11^. Assuming a high degree of symmetry between the two hemispheres, brain volumes were used both in their original orientation and left-right-flipped, effectively doubling the number of input samples in the template creation. Robust averaging was applied to reduce the effect of outliers on the template^12^. First, an initial template was computed as the robust mean of all the volumes along with the Allen CCF template (with 15% weighting). Then, all DAPI volumes were registered to this initial template and a new robust mean computed of the aligned images. This averaging-and-registration process was iterated through three rigid, two affine, and then three non-linear transformations.

We then performed a second round of this entire process, including the stacking of slices to volumes and population-based averaging. In the reconstruction step, the final DAPI template, together with 15% weighting with the Allen CCF template, from the first round was used as a reference, with addition of two further non-linear registration steps (Fig. 1b). The population-based averaging was run with only the reconstructed brains, completely leaving out the Allen CCF template from the final average (Fig. 1c). The last population-based average of the second round is the final three-dimensional reconstruction of the C57BL/6 mouse brain in the DAPI staining modality, which we named *dapi_template.nii.gz*. Note that the final average was interpolated with a three-fold higher resolution in the anterior-posterior axis, yielding a final voxel size of 8.6 × 33.3 × 8.6 µm.

### Brain region segmentation

To create an atlas corresponding to the DAPI template, we nonlinearly registered the Allen CCF template to each of the reconstructed brains. To improve accuracy, both the population average STP modality and the single specimen Nissl staining were used as input images for the registration, with equal weighting. The transformations from these registrations were applied to the Allen segmentation to align the segmentation with each of the reconstructed DAPI-volumes. The segmentations were then transformed to the population-based average space in both the original orientation and flipped in the left-right-axis. For each voxel, a vote-scoring determined the final label in the DAPI-atlas. Finally, the 514 structures of the Allen segmentation were compiled into 11 well-known structures clearly recognizable in the DAPI template (Table 1). Due to the manual mounting of the brain slices on glass slides, the DAPI template exhibited larger ventricles than the CCF, wherefore the ventricle segmentation was manually corrected using the Active Contour-tool in ITK-SNAP 3.2^13^.

**Table 1:** The simplified segmentation regions and the segmentation image value for each region.

Region	Segmentation image value
Background	0
Hippocampus	1
Thalamus	2
Hypothalamus and pallidum	3
Septal complex and fimbria	4
Caudoputamen	5
Dorsal cortex	6
Ventral cortex	7
Lateral cortex	8
Corpus callosum	9
Ventricular system	10
Midbrain	11

### Software

Pre-processing, volume reconstruction, template creation, and automatic segmentation pipelines were implemented in Python 3.6. Registration was performed using ANTs 2.1.0^11^, using either mutual information (MI), cross correlation (CC), or mean squared as similarity metric. Nonlinear registration steps were carried out using the SyN^14^ transformations.

### Animal handling

All treatments and imaging were performed according to protocols approved by the Danish Animal Experiments Inspectorate.

## Data Records

The data set includes the final DAPI template (*dapi_template.nii.gz*) with both the full segmentation based on the Allen atlas containing a total of 514 regions (*dapi_template_segmentation_full.nii.gz*), and with our simplified segmentation of 11 regions (*dapi_template_segmentation_simple.nii.gz*). Moreover, the data set includes the original microscopy images of consecutive coronal brain slices of each mouse brain in tif-format (ex. *BM03_S01.tif*), the pre-processed images in nifti-format (ex. *BM03_S01.nii*), and the reconstructed brain volumes in nifti-format (ex. *BM03.nii*). Each mouse is accompanied with an *ex vivo* MR image of the intact mouse brain before slicing (ex. BM03_MRI.nii), to serve as a reference for the intact anatomy. In Table 2, the mouse identification number, body weight, number of obtained consecutive coronal brain slices, brain length, brain width, and brain volume are listed. The data set can be found in the repository (10.12751/g-node.16wrxa)^15^.

**Table 2:** Animal IDs, weight of mouse in gram, number of consecutive slices acquired from the brain, length of brain, width of brain, and volume of brain. Brain measurements were acquired from the MRI. 12 male C57BL/6 mice aged 10–11 weeks.

Subject IDs	Weight (g)	Nr. of consecutive brain slices	Brain length (mm)	Brain width (mm)	Brain volume (mm^3^)
BM03	24.7	68	14.89	9.458	476.0
BM04	22.5	64	13.06	9.494	439.8
BM06	23.0	62	14.58	9.456	473.0
BM10	26.0	64	14.34	9.570	487.6
BM11	24.0	64	14.73	9.532	479.5
BM12	25.0	64	14.70	9.342	458.8
BM13	27.5	68	14.81	9.604	466.5
BM14	26.5	69	14.51	9.418	425.5
BM15	26.0	69	13.78	9.233	412.3
BM16	26.0	67	13.97	9.266	413.7
BM17	25.8	65	14.00	9.290	432.0
BM18	24.5	70	14.47	9.228	468.0
Mean ± standard deviation	25.1 ± 1.46	—	14.32 ± 0.53	9.41 ± 0.13	452.7 ± 26.70

## Technical Validation

### Visual evaluation, landmark point distances, and coefficient of variance

It has been estimated that brains obtained from 8–10 mice are representative for the population of mice from a given strain in terms of morphometric variance^12^. In this study, we used brains from 12 mice and exploited the symmetric nature of the two hemispheres effectively to yield 24 samples. All brain slices were kept in the correct left-right orientation when handled, which was confirmed by identifying accidental fiducial markers (e.g. tiny surface cuts) and confirming the consistent location of the artefact in neighboring slices.

Visualization of the individual brain reconstruction (Fig. 1b) shows how the iterative reconstruction process produces a smooth brain surface with well-defined structures in the horizontal and parasagittal planes, despite having been reassembled from relatively thick coronal sections. For example, the indentations from the route of the middle cerebral artery and its first branching can easily be identified on the brain surface, and the border between caudoputamen and cortex is smooth and well-defined.

Visual inspection of the final template (Fig. 1c) confirms that the major structures, including the hippocampus, ventricles, caudoputamen, and cortex, are all well-aligned. Smaller structures are easily recognizable and well-defined (e.g. anterior commissure and *corpus callosum*). Furthermore, structures with minimal contrast differences in the single animal DAPI slices, such as the subnuclei of the thalamus, are readily visible in the final template, demonstrating the improved signal-to-noise ratio obtained from multi-sample averaging (Fig. 1c. coronal).

**Fig. 2:**
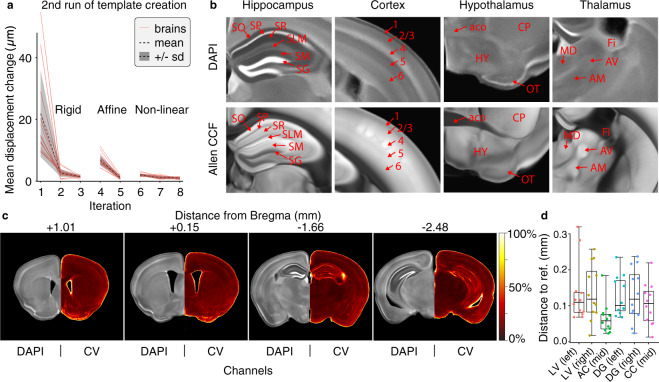
Quantitative landmark point distance validation and qualitatively visual validation of the DAPI template. (**a**) Mean change in displacement between iterations of the second round of the population-based template creation. (**b**) Selected zoom-ins of the hippocampus, cortex, hypothalamus, and thalamus in both the DAPI template and the Allen reference atlas modalities showing the difference in contrast between the two. Regions marked: *stratum oriens* (SO), *stratum pyramidale* (SP), *stratum radiatum* (SR), *stratum lacunosum-moleculare* (SLM), *stratum moleculare* (SM), *stratum granulosum* (SG), somatosensory cortex area, layer 1–6, *hypothalamus* (HY), *anterior commissure, olfactory limb* (aco), *caudoputamen* (CP), *olfactory tubercle* (OT), *mediodorsal nucleus of thalamus* (MD), *anteroventral nucleus of thalamus* (AV), *anteromedial nucleus* (AM), *fimbria* (Fi) (**c**) Representative slices of the DAPI template with corresponding coefficient of variation (CV: standard deviation/mean) maps. (**d**) Distance of the individual landmark points to the reference landmark point for the six positions **LV** (left): lateral ventricle left, **LV** (right): lateral ventricle right, **AC** (mid): anterior commissure midsagittal, **DG** (left): dentate gyrus left, DG (right): dentate gyrus right, **CC** (mid): *corpus callosum* midsagittal.

To test that the population-based template creation converged properly, we plotted the average displacement field change from one iteration to the next during the second round of the template construction (Fig. 2a). For each type of registration (rigid, affine and nonlinear), average displacement change dropped to around 1 µm in the final iteration.

Inspection of the fine structures in the template revealed high contrast in the detailed organization of the zones of the hippocampus, the different layers of the neocortex, the ventral part of the cortex and hypothalamus, and thalamus (Fig. 2b). Specifically, we see more pronounced edges of the hippocampal stratum *lacunosum-moleculare* (SLM) and well-defined edges between layers 4, 5, and 6 in the somatosensory areas of the cortex. These striking differences between the DAPI template and the Allen reference atlas emphasizes the novelty and applicability of the DAPI template to distinguish brain structures based on a cytoarchitectural contrast. Moreover, side-by-side comparisons show the structures of the DAPI template to be well-aligned with the Allen reference template. Figure 2c shows representative slices, along with an image depicting the coefficient of variation (CV; standard deviation/mean) of the image intensity, demonstrating generally low variance in brain structures, but with an expected elevated variance at the edges, most likely due to small biological variability.

To validate quantitatively the co-registration quality of the reconstructed brain volumes in the final template, we identified six landmark points in the raw stack of each brain. Proceeding from anterior to posterior, we marked the following landmarks (position in the anterior-posterior direction in parentheses): the lateral ventricles **LV** (the slice before they open up), the anterior commissure **Ac** (where it connects midsagittally), *corpus callosum***Cc** (where it first connects midsagittally), and the dentate gyrus **Dg** (when the top and bottom granulate layers are equally wide) on each side. The approximate stereotactic coordinates^7^ of each landmark point is shown in Table 3. The landmark points were transformed to the final template space and the distance to landmark points in the template calculated geometrically (Fig. 2d). We found that all the individual landmark points are within a reasonable distance to the template landmark point with a mean distance of approximately 0.1 millimeters. This distance is similar to the thickness of the slices and comparable with that of other studies using landmark point distances^16,17^.

**Table 3:** The stereotactic coordinates of each landmark point in millimetres^7^.

Landmark point	A/P	M/L	D/V
Lateral ventricle, **LV** (left)	1.7	−0.55	4.2
Lateral ventricle, **LV** (right)	1.7	0.55	4.2
Corpus callosum, **CC** (mid)	1.1	0.0	2.8
Anterior commissure, **AC** (mid)	0.1	0.0	4.4
Dentate gyrus, **DG** (left)	−1.6	−0.4	2.2
Dentate gyrus, **DG** (right)	−1.6	0.4	2.2

### Automatic slice segmentation

**Fig. 3:**
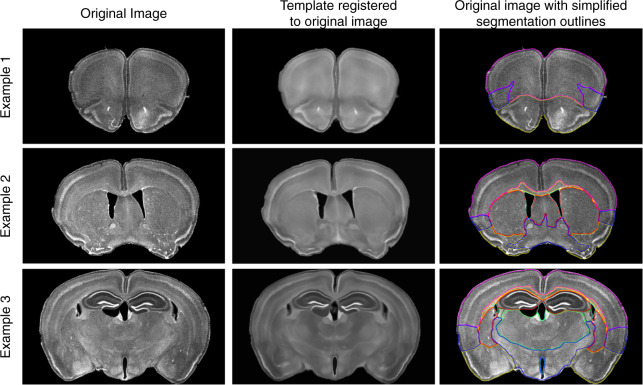
Automatic slice segmentation examples. Three examples of automatic segmentation of new slices, illustrated with outlines of the simplified segmentation. Each example shows the original image (left), the DAPI template fully registered to the new slice (middle), and the original image with the outlines of the simplified segmentation overlaid (right). Example 1 is approximately 2.21 mm from bregma, example 2 is approximately 0.35 mm from bregma, and example 3 is approximately −1.72 mm from bregma.

To test the applicability of the new DAPI template for automatic segmentation upon registration, we acquired three novel brain slices prepared for another study by an investigator not involved in the present study, along with their accompanying coordinates relative to bregma. To allow for minor errors in the bregma positioning, we extracted the corresponding coronal slice and six adjacent slices (three posterior and three anterior) of the DAPI template. Each template slice was rigidly registered to the input slice and the template slice with the highest similarity score (weighted score of 0.7 mutual information and 0.3 Dice coefficient) was selected. The chosen template slice was affinely and nonlinearly registered to the input slice and the transformation was applied to the simplified DAPI template segmentation and displayed as colored outlines (Fig. 3). This procedure successfully registered the DAPI template to the novel slices, demonstrating the feasibility of automatic segmentation of DAPI-stained coronal mouse brain slices.

## Usage Notes

### Automatic slice segmentation

A package of Python scripts is included to automatically spatially normalize and segment novel DAPI-stained coronal mouse brain slices. A high-resolution, DAPI stained.tif image can be provided as input to runner.py along with an approximate bregma-coordinate, the DAPI template, and the template segmentation. This will output a folder containing the automatically generated segmentation of the input slice. An example of usage is provided in the Example folder.

### Visualization with Allen reference atlas

To allow visualization of the Allen Institute reference atlas superimposed on the DAPI template, the repository contains an affine transformation from the Allen reference atlas.nrrd file to the DAPI template space, as well as a guide on how to apply the transformation.

## Data Availability

All the code used for the pre-processing, brain reconstruction, and template creation can be found in the repository (10.12751/g-node.16wrxa) along with the code for automatic slice segmentation.
